# Balanced opioid-free anesthesia with lidocaine and esketamine versus balanced anesthesia with sufentanil for gynecological endoscopic surgery: a randomized controlled trial

**DOI:** 10.1038/s41598-024-62824-3

**Published:** 2024-05-23

**Authors:** Yang Hu, Qing-yun Zhang, Guan-chao Qin, Guo-hong Zhu, Xiang Long, Jin-fei Xu, Yuan Gong

**Affiliations:** Institute of Anesthesiology and Critical Care Medicine, China Three Gorges University and Yichang Central People’s Hospital, Yichang, 443000 Hubei China

**Keywords:** Medical research, Signs and symptoms

## Abstract

In this randomized controlled trial, 74 patients scheduled for gynecological laparoscopic surgery (American Society of Anesthesiologists grade I/II) were enrolled and randomly divided into two study groups: (i) Group C (control), received sufentanil (0.3 μg/kg) and saline, followed by sufentanil (0.1 μg/kg∙h) and saline; and (ii) Group F (OFA), received esketamine (0.15 mg/kg) and lidocaine (2 mg/kg), followed by esketamine (0.1 mg/kg∙h) and lidocaine (1.5 mg/kg∙h). The primary outcome was the 48-h time-weighted average (TWA) of postoperative pain scores. Secondary outcomes included time to extubation, adverse effects, and postoperative sedation score, pain scores at different time points, analgesic consumption at 48 h, and gastrointestinal functional recovery. The 48-h TWAs of pain scores were 1.32 (0.78) (95% CI 1.06–1.58) and 1.09 (0.70) (95% CI 0.87–1.33) for Groups F and C, respectively. The estimated difference between Groups F and C was − 0.23 (95% CI − 0.58 − 0.12; *P* = 0.195). No differences were found in any of the secondary outcomes and no severe adverse effects were observed in either group. Balanced OFA with lidocaine and esketamine achieved similar effects to balanced anesthesia with sufentanil in patients undergoing elective gynecological laparoscopic surgery, without severe adverse effects.

Clinical Trial Registration: ChiCTR2300067951, www.chictr.org.cn 01 February, 2023.

## Introduction

Opioid-free anesthesia (OFA) has been proposed based on the concept of multimodal anesthesia combined with dexmedetomidine, magnesium sulfate, ketamine, lidocaine, and regional block techniques without the need for opioids^1^. However, a previous study reported that OFA administration resulted in a greater incidence of serious adverse events^2^. Thus, there have been great concerns and controversies regarding the safety and benefits of OFA, although its use has been supported by several studies^2,3^.

Lidocaine is a local anesthetic and has been considered a component of multimodal drugs because of its analgesic, anti-hyperalgesic, anti-inflammatory, and immunomodulatory capabilities related to surgical stress^4–6^. Our study demonstrated the successful application of OFA with lidocaine in patients^7^. Esketamine is the s-enantiomer of ketamine and has a higher affinity for N-methyl-D-aspartate receptors than ketamine. Existing evidence indicates that esketamine has great analgesic and anesthetic activities and is widely used for pain management as well as an adjunct to general anesthesia^8–11^.

We hypothesized that OFA with lidocaine and esketamine would exert similar anesthetic effects and provide better perioperative analgesia. The 48-h time-weighted average (TWA) of the numeric rating scale (NRS), chosen as the primary outcome, was used to evaluate clinical benefits.

## Materials and methods

### Study design

This was a single-center, double-blind, parallel-group, randomized controlled trial. Ethical approval was obtained from the Institutional Review Board of the Yichang Central People’s Hospital (HEC-KYJJ-2022-077-02). This study was conducted in compliance with the tenets of the Declaration of Helsinki and was registered in the Chinese Clinical Trials Registry (ChiCTR2300067951).The trail was registered on 1 Feberary, 2023,and were conducted from 10 Feberary to April 30, 2023. Written informed consent was obtained from all patients, and the study complied with the Consolidated Standards of Reporting Trials checklist.

### Patients

The inclusion criteria were as follows: women aged 18–65 years, American Society of Anesthesiologists grade I or II, and scheduled for elective laparoscopic gynecological surgery. The exclusion criteria were as follows: an untreated underlying disease (e.g., hypertension, epilepsy, and diabetes), body mass index (BMI) ≥ 30 kg/m^2^, hemoglobin concentration ≤ 80 g/l, and severe cardiac arrhythmias (e.g., second-II-II atrioventricular block, atrial fibrillation, and heart failure). Patients who refused to participate in the study were also excluded.

### Primary outcomes

The primary outcome was the 48-h TWA of the NRS^12^**.** The time points of the NRS assessment were as follows: within 10 min after tracheal extubation (T_0_) and 1 h (T_1_), 4 h (T_2_), 12 h (T_3_), 24 h (T_4_), and 48 h (T_5_) after surgery.

### Secondary outcomes

The secondary outcomes included the time for extubation and sedation scores at 0, 30, and 60 min after extubation (Richmond Agitation-Sedation Rating Scale, RASS)^13^. The time of the first bowel sound and time of bowel ventilation were recorded. Adverse events (pruritus, insomnia, metallic taste, nausea, vomiting, and psychiatric symptoms) were recorded and analyzed. Analgesic consumption over 48 h postoperatively was recorded and converted to morphine equivalents, based on previous studies^14,15^.

### Randomization and interventions

A computer-generated randomized sequence was created by an independent investigator, Jin-fei Xu, who was blinded to the study, using SPSS 22.0 (IBM Corporation, Armonk, NY, USA) in a 1:1 allocation. The patients were randomly divided into two groups (Group F [OFA] and C [control]), with each patient assigned a randomized number.

### Perioperative anesthetic care

Patients fasted for 6 h and were restrained with oral intake of clear fluids for 2 h perioperatively. Non-invasive blood pressure (BP), heart rate (HR), electrocardiography, and pulse oxygen saturation were measured using a multifunction monitor (GE Healthcare, Helsinki, Finland) when the patients entered the operating room. Baseline data were obtained from the average of three measurements obtained 2 min apart. A 20 G intravenous catheter was placed in the peripheral vessels of the left forearm.

The balanced opioid-free anesthesia using the lidocaine and esketamine protocol was administered as follows: lidocaine (1.5 mg/kg) and esketamine (0.15 mg/kg) were infused over 5 min in Group F. Additionally, the standard general anesthesia protocol included the infusion of sufentanil (0.3 μg/kg) and saline over 5 min in Group C. Following the initial infusion, propofol (2 mg/kg) and rocuronium (0.6 mg/kg) were injected, followed by the continuous infusion of lidocaine (2 mg/kg∙h) and esketamine (0.10 mg/kg∙h) in Group F, and sufentanil (0.1 µg/kg∙h) and saline at the same rate in Group C.

Thereafter, 500 mL of Ringer’s lactate solution was injected after the catheter setup. The anesthesiologist opened a sealed envelope containing the computer-generated randomized assignment number before induction. An anesthesia nurse blinded to the study prepared and activated the infusion of study drugs following the study protocol. They were instructed to reveal the randomized assignment to the patients or study co-investigators. Anesthesia was maintained with sevoflurane (minimum alveolar concentration 0.8–1.2) and 50% oxygen in medical air in both groups. Vasopressors were administered to maintain the mean arterial BP within ± 20% of baseline measurements. Before extubation, all patients received 4 mg betamethasone, 5 mg tropisetron, and 50 mg flurbiprofen axetil as postoperative analgesia. An anesthesiologist blinded to the study performed anesthesia emergence, assessed, and recorded the time from the end of surgery to extubation, sedation scores, and NRS scores.

The patients were monitored for at least 5 min to resume regular spontaneous respiration and subsequently transferred to the post-anesthesia care unit (PACU). An anesthesia nurse (Guo-hong Zhu) blinded to the study visited the patients and used the RASS to evaluate them at 0, 30, and 60 min after their transfer to the PACU until the score returned to 0. Metoclopramide (1 g) and ondansetron (4 mg) were used as rescue medicines for postoperative nausea and vomiting (PONV). Postoperative pain assessment was conducted by an anaesthetist (Xiang Long), who was blinded to the study, at 10 min after tracheal extubation (T_0_) and 1 h (T_1_), 4 h (T_2_), 12 h (T_3_), 24 h (T_4_), and 48 h (T_5_) after surgery using Numeric Rating scale (NRS). The anesthetist, Xiang Long, recorded the time to the first bowel sounds after surgery.

The postoperative pain management protocol consisted of 30 mg/8 h ketochromic acid injections and patient-controlled intravenous analgesia (PCIA). The protocol of PCIA was as follows: sufentanil 100 µg, nalbuphine 40 mg, with saline to 225 ml (the infusion rate was set up at a bolus dose of 3 ml/h, and lockout period of 15 min, max 3 dose/h, with no background infusion). Nalbuphine is considered a rescue analgesic in the PACU. Patients were assessed for discharge from the PACU to the ward according to the institution’s standards, where they remained overnight. An experienced investigator, Jin-fei Xu, who was blinded to the study, initiated the process of recording the need for analgesics within 48 h after surgery and the incidence of PONV during the 48 h postoperative period. Perioperative care followed the practices of local clinicians. Patient satisfaction was assessed using a 5-point scale, with 0 indicating "unsatisfactory" and 5 indicating "very satisfied", and was recorded 48 h after surgery.

### Statistical analysis

Based on the preliminary experimental results, the sample size calculations achieved a power of 90% with *α* = 0.05 to reject the null hypothesis. Considering that approximately 10% of patients were lost to follow-up, 74 were scheduled for the study. Age, height, weight, BMI, time of surgery, time to provide anesthesia, time to extubate, NRS score, consumption of analgesics, and time for recovery of intestinal function is expressed as mean ± standard deviation and were analyzed using Student’s *t*-test. RASS scores and patient satisfaction were analyzed using the Mann–Whitney U-test. Inter-group comparison was conducted using the T-test of two independent samples. *T*-tests for independent samples were used to compare post-operative analgesic consumption between the groups. Moreover, the pain scores (NRS values) at 0, 1, 4, 12, 24, and 48 h postoperatively were analyzed using an independent Student’s *t*-test for two independent samples. The incidence of PONV and adverse effects were analyzed using the χ^2^ test. Statistical significance was set at *P* < 0.05. SPSS version 22 (SPSS, Chicago, IL, USA) was used to perform the statistical analyses.

## Results

### Patient characteristics

Overall, 74 patients were enrolled in the study, of whom 72 were included in the analysis. One patient in each group was excluded from the study. No statistically significant differences were observed in the baseline data between the groups, except for age (Group F (40.83 [9.03]) vs. Group C (45.64 [10.13]), *P* = 0.037). There were no statistical differences in surgery duration (Group C [91.86 (39.5) min] vs. Group F [99.47 (47.8) min, (*P* = 0.422)]) and time of anesthesia (Group C [119.81 (40.78) min] vs. Group F [126.78 (48.84) min) (*P* = 0.551)]). However, there was a statistically significant difference in the time to extubation (8.50 [2.51] min in Group C vs. 10.39 [2.16] min in Group F, *P* = 0.013) (Table 1).

**Table 1:** Baseline characteristics of the participants.

	Group C (*n* = 36)	Group F (*n* = 36)	*P*-value
Age (years)	45.64(10.13)	40.83(9.03)	0.037
Height (cm)	158.67(4.02)	159.97(3.86)	0.164
Weight (kg)	57.63(9.99)	60.33(8.96)	0.230
BMI (kg/m^2^)	23.40(2.78)	23.53(2.89)	0.853
ASA (I/II)	23/13	19/17	0.339
Surgery duration (min)	91.86(39.5)	99.47(47.8)	0.422
Anesthesia duration (min)	119.81(40.78)	126.78(48.84)	0.551
Time to extubation (min)	8.50(2.51)	10.39(2.16)	0.013

Continuous variables are expressed as means and standard deviations, and categorical variables are expressed as (+ /−). *P*-values < 0.05 indicate significant differences. *ASA* American Society of Anesthesiologists, *BMI* Body mass index.

### Forty-eight-hour TWAs of postoperative NRS scores

**Figure 1:**
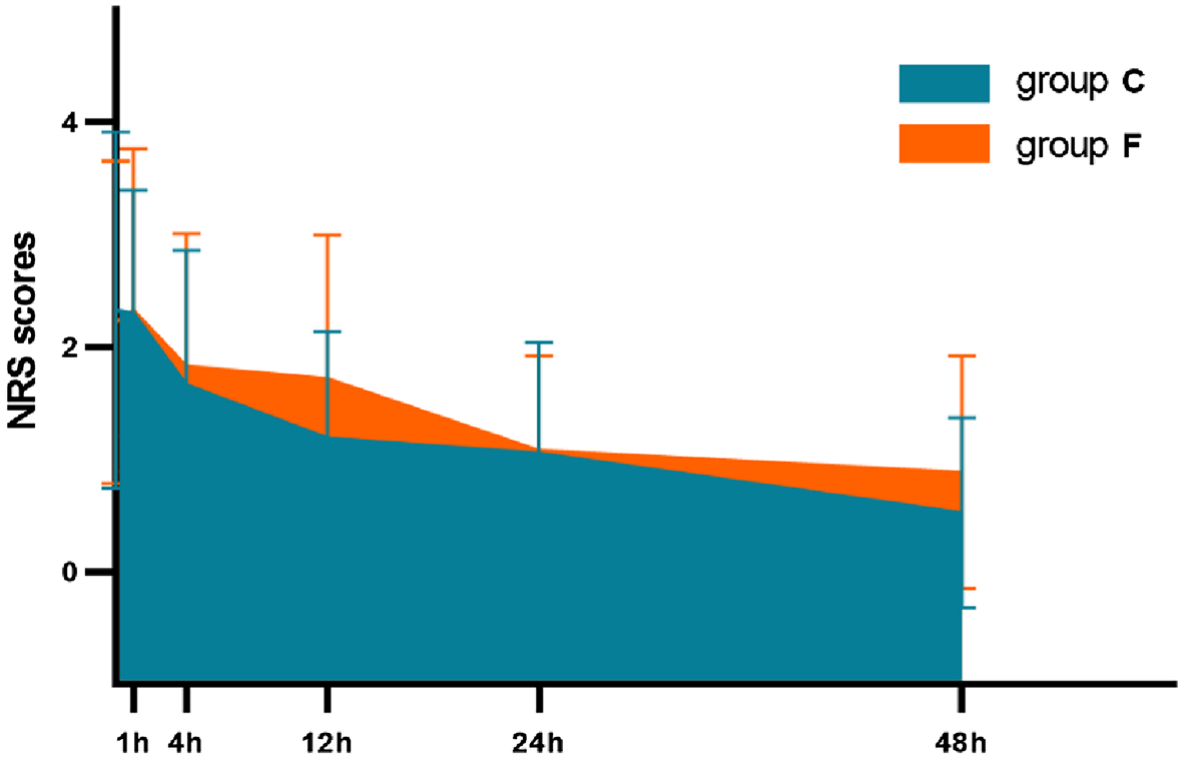
Time-weighted average of NRS scores. *NRS*: numeric rating scale (1–10), *TWA*: time-weighted average during 48 h postoperatively, variables are expressed as means ± SD (standard deviations).

The mean (SD) values of the NRS scores at different time points during the 48-h postoperatively were set as line plots, and the area under the curve was calculated and expressed as the TWA. The results were 1.32 (0.78) and 1.09 (0.70) for Groups F and C, respectively. The estimated difference between Groups F and C was − 0.23 (97.5% CI − 0.58 − 0.12; *P* = 0.195). However, we believe that this difference was clinically insignificant. Table 2 summarizes these results (Fig. 1).

**Table 2:** NRS scores at each time-point and the 48-h time-weighted pain score.

	Group C (*n* = 36)		Group F (*n* = 36)		*P*-value
	Mean/SD	95% CI	Mean/SD	95% CI	
NRS-PACU	2.333 (0.252)	(1.830, 2.836)	2.222 (0.252)	(1.719, 2.725)	0.756
NRS-1 h	2.306 (0.212)	(1.882, 2.729)	2.333 (0.212)	(1.910, 2.757)	0.927
NRS-4 h	1.667 (0.198)	(1.271, 2.062)	1.833 (0.198)	(1.438, 2.229)	0.554
NRS-12 h	1.194 (0.188)	(0.820, 1.569)	1.722 (0.188)	(1.348, 2.097)	0.051
NRS-24 h	1.056 (0.153)	(0.751, 1.360)	1.083 (0.153)	(0.779, 1.338)	0.898
NRS-48 h	0.528 (0.158)	(0.214, 0.842)	0.889 (0.158)	(0.575,1.203)	0.110
TWA-48 h	1.09 (0.70)	(0.87,1.33)	1.32 (0.78)	(1.06,1.58)	0.195

Continuous variables are expressed as means and standard deviations.

*NRS* Numeric rating scale (1–10), *PACU* Post-anesthesia care unit, *TWA* Time-weighted average during 48 h postoperatively; *P*-values < 0.05 indicate significant differences.

### Pain score at 48-h postoperatively

There were no differences in pain scores between the groups at T0, T1, T2, T3, T4, and T5 (Supplementary Figure S1). No statistical differences were observed between Groups C and F at the different time points (Table 2).

### Postoperative analgesic consumption at 48-h

The postoperative analgesic consumption was 0.832 (0.455) mg/kg in Group C and 0.788 (0.267) mg/kg in Group F. There was less analgesic consumption in Group F, which was not statistically different from that in Group C.

### PONV

Twelve patients in Group C and 10 patients in Group F experienced nausea and vomiting 48 h postoperatively (33.3% in Group C vs. 27.8% in Group F, *P* = 0.609). No significant differences were observed among the study groups. We did not observe any other adverse effects.

### Richmond agitation–sedation scale scores

**Figure 2:**
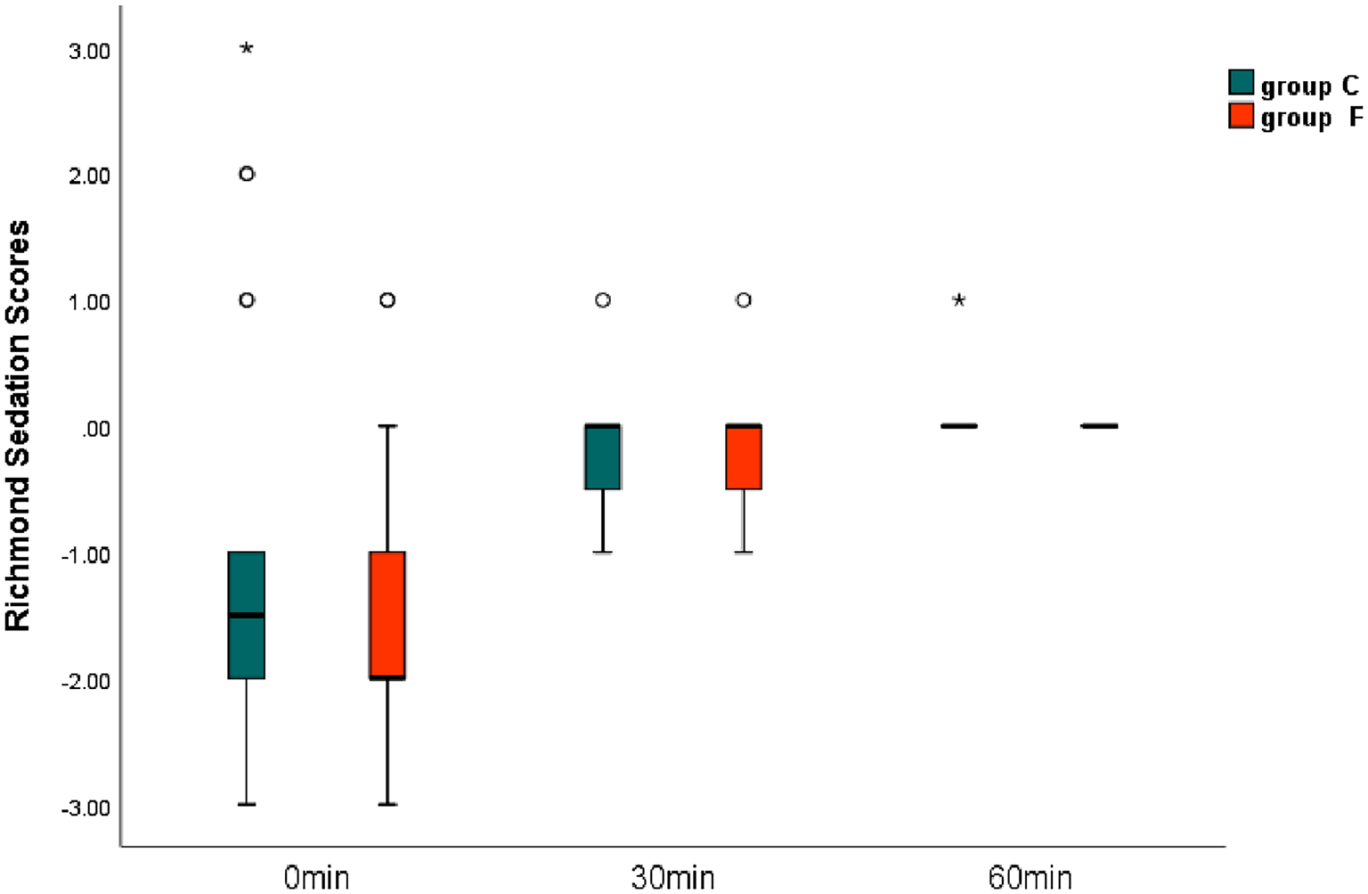
The Richmond Sedation Scores. Variables are expressed as means ± SD (standard deviations).

The sedation score at 0 min after transfer to the PACU was − 2 (− 2, − 1) in Group C and − 2 (-2, − 1) in Group F (*P* = 0.219). At 30 min after transfer to the PACU, it was 0 (− 0.75, 0) in Group C and 0 (0.75, 0) in Group F (*P* = 1.0), and at 60 min after transfer to the PACU, it was 0 (0,0) in Group C and 0 (0,0) in Group F (*P* = 0.317) (Fig. 2).

### Postoperative gastrointestinal recovery

There were no significant differences in the time to first bowel sounds (13.027 [5.893] h in Group C vs. 14.536 [4.768] h in Group F, *P* = 0.237) between the groups and time to first intestinal exhaust (20.306 [4.780] h in Group C vs. 20.556 [5.127] h in Group F, *P* = 0.831).

### Patient satisfaction score

There was no statistical difference between the two groups in patient satisfaction score, with a median of 5 (4–5) (“very satisfied”) and 4 (4–5) (“satisfied”) in Groups F and C, respectively (*P* = 0.29).

## Discussion

In our trial, balanced OFA with lidocaine and esketamine resulted in similar effects as balanced opioid anesthesia in peri-and post-operative analgesia consumption within 48 h. No statistical differences were observed between the groups in terms of NRS scores and gastrointestinal recovery. Patients in Group F had lower RASS scores, time for extubation and similar patient satisfaction scores. More importantly, no severe adverse events were observed during the trial.

Appropriate perioperative pain control is essential to facilitate postoperative patient recovery. Perioperative pain control remains over-reliant on opioids due to their fast onset time and strong analgesic effect, even though they have significant adverse effects, and there is great controversy regarding the effectiveness and safety of OFA^16–19^. Our study found that the 48-h TWA NRS scores and postoperative NRS scores at different time points were consistently lower in both groups (Supplementary Table S1). We also found that OFA with lidocaine and esketamine in patients achieved similar peri- and postoperative analgesia consumption as balanced anesthesia with sufentanil, which was similar to previous studies^3,16^. The reason may be attributed to the following: (i) multimodal analgesia was used; (ii) lidocaine could reduce postoperative pain^20^; and (iii) esketamine has a better analgesic effect, which was used as an adjuvant for perioperative analgesia.

Postoperatively, gastrointestinal dysfunction resulted in a longer hospital stay and poorer hospital experience. As previously mentioned, better perioperative analgesia, including that for gastrointestinal function, can promote postoperative recovery. However, opioids can disturb gastrointestinal function via their receptors in the gastrointestinal tract^21^. Thus, OFA can be used to treat postoperative gastrointestinal dysfunction. Our study found that the time to the first bowel sound in Group F was nearly 1.5 h earlier than that in Group C, despite no significant differences between the groups. The reduction in the time to first bowel sounds showed the positive effects of OFA. However, this may be because gastrointestinal function recovery was not chosen as the primary outcome, which may have affected the calculation of the sample size.

The administration of opioids is a risk factor for PONV, which is known to have a high incidence after surgery^22^. The mechanism is multifactorial, involving (a) enhanced vestibular sensitivity (symptoms may include vertigo and worsening with motion), (b) direct effects on the chemoreceptor trigger zone, and (c) delayed gastric emptying (symptoms of early satiety and bloating, worsening postprandially)^22^, where the distribution of opioid receptors in gastrointestinal tracts is not confined to the colon, but also higher segments^23^. However, we found no statistical difference between the groups, even with a lower incidence of PONV in Group F, which is similar to the results of a previous study^24^. Moreover, the 48-h TWA of pain scores served as the primary outcome and resulted in a small sample size when other analyses were performed.

Delay in extubation is commonly associated with increased adverse outcomes^25^.Anesthetic drugs are one element affecting the time of extubation, and a study demonstrated that the time for extubation was prolonged with opioid administration^26^. Our trail yielded a similar result, with extubation time being 2 min longer in group F. The reasons maybe be: (1) opioid resulted in respiratory depression; (2) the analgesic effect of opioids reduced pain caused by endotracheal catheters.

Another side-effect of opioid administration is sedation^27^. A previous study showed that balanced OFA resulted in prolonged sedation than balanced opioid anesthesia^2^. We used RASS scores to assess post-anesthesia sedation at 0, 30, and 60 min after entering the PACU during our trial. No differences were observed at each time point between groups, and most patients demonstrated a better sedation state (RASS scores = 0) at 30 min in the PACU. There was no difference between groups in patient satisfaction scores at 24 h postoperatively. We believe this is because OFA with lidocaine achieves a better quality of recovery during general anesthesia^7^; esketamine has hypnotic, analgesic, and sympathomimetic effects that reduce the requirement of individual drugs and minimize sedation effects^28,29^.

Considering the side effects of lidocaine (toxicity and allergic reaction) and ketamine (itching, mental confusion, and nightmares)^30,31^, we did not observe such severe side effects during our trial, which is different from a previous study that was terminated for severe adverse events (bradycardia) during the trial^2^. This may have occurred due to the different combinations of drugs for OFA, especially with the administration of dexmedetomidine, which causes bradycardia as one of its main side-effects. Regarding the side effects of dexmedetomidine, we did not choose it as an adjunct, and no severe bradycardia was found during the study. Another reason maybe that we administrated lidocaine and esketamine within a safe range.

Our study had some limitations. First, it was a single-center study, which may have limited its efficacy. Second, the surgery type was gynecological endoscopic surgery; more studies need to be performed on other surgical types. Third, we chose opioids for postoperative analgesia, which may have affected the assessment of postoperative analgesia.

In conclusion, balanced OFA with lidocaine and esketamine achieved similar effects to balanced opioid anesthesia without severe side effects. Further studies are needed to determine the effects and safety of OFA.

### Supplementary Information

Supplementary Table 1.

Supplementary Figure 2.

## Data Availability

Individual de-identified participant data (including data dictionaries) will be shared; All of the individual participant data collected during the trail, after deidentification; Study protocol,stastical analysis plan, informed consent form, clinical study report, analytic code will be available; the data will become available immediately following publication and for no end date; Access criteria data will be shared (including with anyone who wishes to access the data for any purpose, and proposal should be directed to gy-yc@163.com.
