# Supplementary material for: Balanced opioid-free anesthesia with lidocaine and esketamine versus balanced anesthesia with sufentanil for gynecological endoscopic surgery: a randomized controlled trial
Source: Sci Rep. 2024 May 23;14:11759. doi: 10.1038/s41598-024-62824-3 (PMC11116438; doi:10.1038/s41598-024-62824-3)
Supplement: Supplementary file 1 — Supplementary Table 1. [file 41598_2024_62824_MOESM1_ESM.docx]

| TABLE S1: NRS scores | | | | | | |
| --- | --- | --- | --- | --- | --- | --- |
|  | |  | deviation | Standard error | 95% CI | |
|  |  |  |  |  | lower limit | upper limit |
| NRS PACU | N | 72 | 0 | 0 | 72 | 72 |
|  | MEAN | 2.2778 | -.0042 | .1744 | 1.9444 | 2.6246 |
|  | SD | 1.50326 | -.01347 | .11978 | 1.25586 | 1.73041 |
| NRS1h | N | 72 | 0 | 0 | 72 | 72 |
|  | MEAN | 2.3194 | -.0049 | .1464 | 2.0278 | 2.6111 |
|  | SD | 1.26520 | -.02712 | .17228 | .93942 | 1.59199 |
| NRS4h | N | 72 | 0 | 0 | 72 | 72 |
|  | MEAN | 1.7500 | -.0028 | .1392 | 1.4722 | 2.0417 |
|  | SD | 1.18381 | -.01199 | .10432 | .96739 | 1.36922 |
| NRS12h | N | 72 | 0 | 0 | 72 | 72 |
|  | MEAN | 1.4583 | -.0058 | .1333 | 1.1944 | 1.7083 |
|  | SD | 1.14986 | -.01441 | .08793 | .96161 | 1.30174 |
| NRS24h | N | 72 | 0 | 0 | 72 | 72 |
|  | MEAN | 1.0694 | -.0048 | .1078 | .8611 | 1.2778 |
|  | SD | .90890 | -.01880 | .12331 | .67822 | 1.14978 |
| NRS48h | N | 72 | 0 | 0 | 72 | 72 |
|  | MEAN | .7083 | .0002 | .1153 | .4861 | .9444 |
|  | SD | .95589 | -.01426 | .10511 | .73145 | 1.13519 |
|  | | | | | | |

Continuous variables are expressed as means and standard deviations.

NRS: Numerical Pain Score (1–10), TWA: time-weighted average during 48 h postoperatively,
