# Supplementary material for: Balanced opioid-free anesthesia with lidocaine and esketamine versus balanced anesthesia with sufentanil for gynecological endoscopic surgery: a randomized controlled trial
Source: Sci Rep. 2024 May 23;14:11759. doi: 10.1038/s41598-024-62824-3 (PMC11116438; doi:10.1038/s41598-024-62824-3)
Supplement: Supplementary file 2 — Supplementary Figure 2. [file 41598_2024_62824_MOESM2_ESM.pdf]

Figure s1 NRS scores at different timepoints during 48h postoperatively

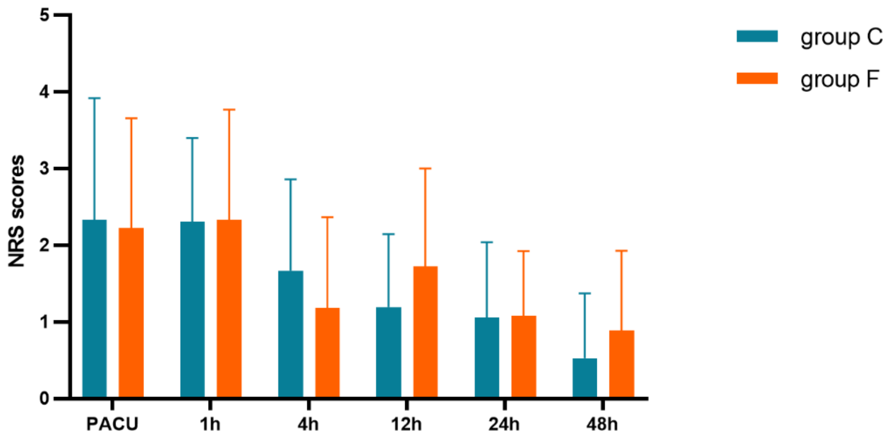

variables are expressed as means±SD(standard deviations).NRS: Numerical Pain Score (1–10), p-values <0.05, indicate statistical differences.
